# B-Mode Ultrasound, a Reliable Tool for Monitoring Experimental Intracerebral Hemorrhage

**DOI:** 10.3389/fneur.2021.771402

**Published:** 2021-12-23

**Authors:** Mari Carmen Gómez-de Frutos, Iván García-Suárez, Fernando Laso-García, Luke Diekhorst, Laura Otero-Ortega, María Alonso de Leciñana, Blanca Fuentes, María Gutiérrez-Fernández, Exuperio Díez-Tejedor, Gerardo Ruíz-Ares

**Affiliations:** ^1^Neurological Sciences and Cerebrovascular Research Laboratory, Department of Neurology and Stroke Center, Neuroscience Area of IdiPAZ Health Research Institute, La Paz University Hospital, Universidad Autónoma de Madrid, Madrid, Spain; ^2^Department of Emergency Service, San Agustín Hospital, University of San Agustin, Asturias, Spain

**Keywords:** B-mode ultrasound, experimental, intracerebral hemorrhage, magnetic resonance imaging, ultrasound, rat

## Abstract

**Background:** Magnetic resonance imaging (MRI) is currently used for the study of intracerebral hemorrhage (ICH) in animal models. However, ultrasound is an inexpensive, non-invasive and rapid technique that could facilitate the diagnosis and follow-up of ICH. This study aimed to evaluate the feasibility and reliability of B-mode ultrasound as an alternative tool for *in vivo* monitoring of ICH volume and brain structure displacement in an animal model.

**Methods:** A total of 31 male and female Sprague-Dawley rats were subjected to an ICH model using collagenase-IV in the striatum following stereotaxic references. The animals were randomly allocated into 3 groups: healthy (*n* = 10), sham (*n* = 10) and ICH (*n* = 11). B-mode ultrasound studies with a 13-MHz probe were performed pre-ICH and at 5 h, 48 h, 4 d and 1 mo post-ICH for the assessment of ICH volume and displacement of brain structures, considering the distance between the subarachnoid cisterns and the dura mater. The same variables were studied by MRI at 48 h and 1 mo post-ICH.

**Results:** Both imaging techniques showed excellent correlation in measuring ICH volume at 48 h (*r* = 0.905) and good at 1 mo (*r* = 0.656). An excellent correlation was also observed in the measured distance between the subarachnoid cisterns and the dura mater at 1 mo between B-mode ultrasound and MRI, on both the ipsilateral (*r* = 0.870) and contralateral (*r* = 0.906) sides of the lesion.

**Conclusion:** B-mode ultrasound imaging appears to be a reliable tool for *in vivo* assessment of ICH volume and displacement of brain structures in animal models.

## Introduction

Intracerebral hemorrhage (ICH) accounts for 10–15% off all strokes (1, 2) and is the most lethal form of stroke, with high morbidity and mortality (3–5). One of the most important prognostic factors is the severity of bleeding and the volume of the hematoma (5).

Due to its high sensitivity and specificity, computed tomography (CT) is the gold-standard for diagnosing ICH in patients in the acute setting (1, 3), whereas magnetic resonance imaging (MRI) is often used as a complementary method to determine the underlying causes and to add information on the evolutionary stage of the ICH (3, 6, 7). In animal models, MRI is the preferred imaging technique to study the brain *in vivo*, particularly in small animals, due to its sensitivity and high-resolution for small animal imaging (8–10). In rat models of ICH, MRI has only recently been employed (8). However, factors such as availability, tolerability, acquisition time, transport and clinical status can hinder the performance of this technique and should be taken into account (3, 11).

Conversely, ultrasound is a rapid, non-invasive, inexpensive, accessible and well-tolerated imaging technique that can be easier to perform (12, 13). Its versatility and ability to obtain dynamic and real-time images allow us to acquire structural and functional information with sufficient spatial and temporal resolution (14). These characteristics make this tool suitable to be used routinely in rodents. Until now, however, although ultrasound has been used to study hemodynamic changes after ICH in animal models, B-mode ultrasound has not been used to assess the brain parenchyma or the structural changes produced by the ICH (15–17).

The development of methods for rapid detection and accurate monitoring of spontaneous ICH in animals is one of the preclinical research priorities of the Hemorrhagic Stroke Academia Industry (HEADS) recommendations (18). Given the above-mentioned advantages, B-mode ultrasound images could be used to assess the structural changes resulting from ICH, helping reduce the use of MRI. Therefore, the aim of this study was to assess the usefulness of B-mode ultrasound imaging for monitoring ICH in an experimental animal model by comparing the data obtained by MRI and ultrasound.

## Materials and Methods

### Animals and Intracerebral Hemorrhage Induction

A total of 33 male and female Sprague Dawley rats (8–9 weeks old, weighing 225–275 g) were employed in this study. We induced general anesthesia with 8% sevoflurane in a 1-l/min oxygen flow and maintained it with 4% sevoflurane in a 1-l/min oxygen flow with a face mask. Meloxicam (2 mg/kg) was subcutaneously administrated for analgesia induction. For ICH induction, the animals were placed in a stereotactic frame (Stoelting). We injected 0.5 U of collagenase type-IV (Sigma-Aldrich, USA) diluted in 1 μL of saline by a craniotomy performed close to the bregma with the following stereotaxic coordinates: 0.04 mm posterior, 0.35 mm lateral and 0.6 mm ventral, as previously described (19). This model is simple and has high reproducibility of striatal ICH (20).

**Figure 1:**
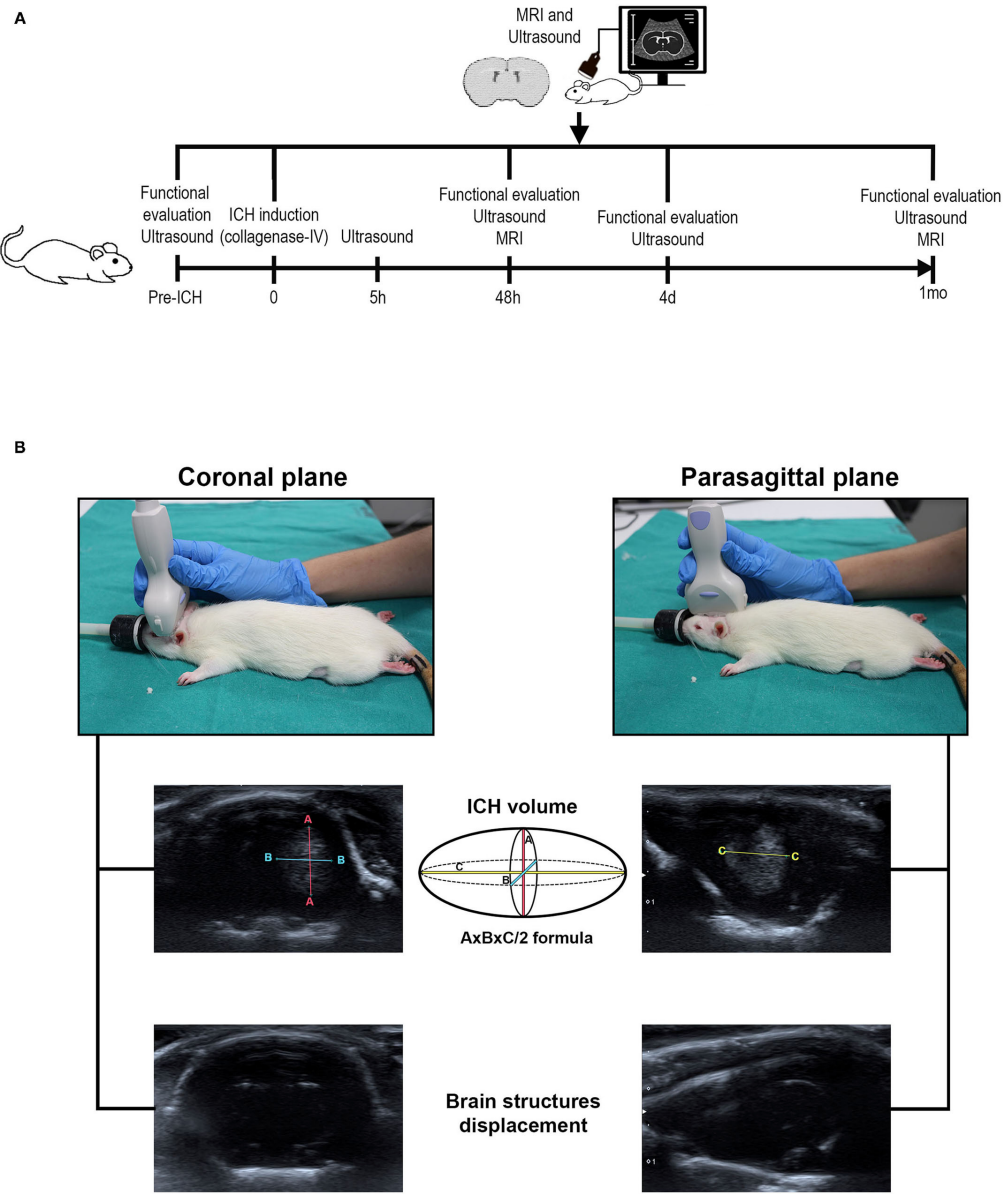
**(A)** Scheme of the experimental animal protocol. ICH was induced in male and female Sprague-Dawley rats by administration of collagenase type-IV in the striatum. Motor function was evaluated by the Rogers, beam walking, tapered beam walking and Rotarod tests pre-ICH and at 5 h, 48 h, 4 d and 1 mo before ICH induction. Imaging studies by MRI (48 h and 1 mo post-ICH) and B-mode ultrasound (pre-ICH and at 5 h, 48 h, 4 d and 1 m post-ICH) were performed to study the volume of hemorrhage as well as the displacement of brain structures. **(B)** Placement of the transducer for B-mode ultrasound studies. The coronal view positioning the transducer perpendicular to the head of the animals allows to study the maximum length “A” and the maximum width “B” of the hemorrhage volume and to analyze brain structures displacement (left image). The thickness of the hemorrhage “C” was acquired in the parasagittal view with the transducer longitudinal to the head (right image). ICH, intracerebral hemorrhage; MRI, magnetic resonance imaging.

The animals were randomly distributed in 3 experimental groups: 1, healthy group (*n* = 5 males and 5 females); 2, sham group (subjected to surgery without hemorrhage, *n* = 5 males and 5 females); 3, ICH group (subjected to hemorrhage, *n* = 6 males and 5 females) (Figure 1A).

### Functional Evaluation Scales

The animals were evaluated before ICH induction and at 48 h, 4 d and 1 mo post-ICH by a researcher blinded to the different experimental groups. The motor function of the animals was studied using the rotarod, beam walking, modified beam walking and modified Rogers tests. All the animals received pre-training 3 d before hemorrhage induction.

The beam walking test studies the capacity of animals to stay on a wooden beam (2.5 × 2.5 × 80 cm), assigning the following scores: 0, crosses the beam without foot slip; 1, crosses but holds on to the lateral side of the beam; 2, able to traverse the beam, but with difficulty, crawling; 3, requires >10 s to cross; 4, unable to cross; 5, unable to move the body or any limb on the beam; 6, unable to stay on the beam >10 s (21).

With the tapered walking beam test, we analyzed the hind limb functions, studying the ability of the rats to cross to another wooden beam. The left hind limb slip ratio was calculated as follows: (total slips + 0.5 × half slips)/total steps × 100% (22, 23).

In order to study the motor coordination of the animals, the rotarod test was performed. The animals were placed in a rotated cylinder that progressively increased in speed (4–40 rpm) for 2 min maximum (24).

A variant of the Rogers test was used to assign scores as follows: 0, no functional deficit; 1, lack of full extension of the forepaw; 2, decreased grip of forelimb while tail gently pulled; 3, spontaneous movement in all directions, contralateral circling if the tail is pulled; 4, circling; 5, movement only when stimulated; 6, unresponsive to stimulation with a decreased level of consciousness; 7, dead (25).

### Imaging

To assess hemorrhage volume and brain structure displacement, both B-mode ultrasound imaging and MRI were performed.

Transcranial B-mode ultrasound (Xario 200G, TUS-X200, Canon) was performed using a 13-MHz lineal multi-frequency transducer (PLU-1204BT, Canon) with a mechanical index of 1.4 and optimization and constant adjustments in gain and depth throughout the experiments. Animals were anesthetized with 8% sevoflurane in a 1 l/min oxygen flow and maintained it with 4% sevoflurane in a 1 l/min oxygen flow with a face mask. Each scanning session had a maximum duration of 10 min.

B-mode ultrasound was performed within 5 h after surgery and at 48 h, 4 d and 1 mo post-ICH to analyze ICH volume. The displacement of brain structures was also measured before ICH induction (pre-ICH). To study the ICH volume, 2 ultrasound scans were performed for each animal, 1 in the coronal plane and 1 in the sagittal plane with the animals placed in a prone position. The transducer was positioned perpendicular to the head to obtain coronal images, and parasagittal images were obtained with the transducer longitudinal to the head (Figure 1B). ICH was identified with hyperechogenic appearance at 48 h and hypoechoic appearance at 1 mo. To study the displacement of the cerebral structures, the subarachnoid cisterns were used as a reference (26). For this purpose, the transducer was placed perpendicular and caudal to the head.

MRI was performed at 48 h and 1 mo post-ICH on a Bruker Pharmascan Biospect system (Bruker Medical Gmbh, Ettlingen, Germany), using T2-weighted (T2-W) spin-echo anatomical images acquired using a 7.0-T horizontal-bore superconducting magnet with a ^1^H circular polarized volume coil with an inner diameter of 40 mm and a Bruker gradient insert 90 mm in diameter (maximum intensity 36 G/cm), equipped with a ^1^H receive-only mouse brain surface coil, volume transmission coil and Bruker gradient insert 90 mm in diameter (maximum intensity 36 G/cm). Animals were anesthetized with a 2% sevoflurane-oxygen mixture in an induction chamber, and the flow of anesthetic gas was constantly regulated to maintain a breathing rate of 50 +/– 20 bpm. The physiological state of the rats was monitored using a monitoring system by SA Instruments (Stony Brook, NY) that controlled the respiratory rate and body temperature. T2 imaging lasted 20 min. Images were analyzed with the ImageJ 1.52 program (National Institutes of Health, USA), identifying the hyperintense lesion at 48 h and the heterogeneous lesion (hyper- and isointense) at 1 mo.

For both the B-mode ultrasound and MRI studies, the AxBxC/2 formula (27, 28) was employed for the ICH volume, and brain structure displacement was studied using as reference the distance between the subarachnoid cisterns and the dura mater previously identified (26). In order to identify the displacement and magnitude of this, the distance from the dura mater to the subarachnoid cisterns was measured, and the cistern displacement ratio (CDR), to normalize and refer to the contralateral side, was calculated with the following formula: cisterns to dura mater distance on the side ipsilateral to the lesion/cisterns to dura mater distance on the side contralateral to the lesion. After normalization of the distance between the subarachnoid cisterns and the dura mater, a value of 1 in the CDR indicates no displacement of brain structures, >1 an increase of the distance in the hemisphere ipsilateral to the lesion, and <1 an increase of the distance in the hemisphere contralateral to the lesion.

### Statistics

The results are expressed as mean ± standard deviation. At least 10 rats were estimated to be required for each group to obtain differences between groups for a significance level of 5% (alpha) and a power of 80% (1-beta). The rats that died before the end of the study were immediately replaced by new ones until a total of 10 rats per group was reached. The data were compared using an analysis of variance for each factor and corrected with Tukey's *post hoc* test when the data followed a normal distribution. If the data followed a non-normal distribution, they were compared with the Kruskal-Wallis test followed by the Mann-Whitney test. In the case of comparisons within the same experimental group, a *t*-test for related samples was performed in the case of normality, and the Wilcoxon signed-rank test in the case of non-normality. Pearson's (*r*) (parametric test) or Spearman's (ρ) (non-parametric test) correlation coefficient was used to measure the strength of the relationships between the variables (functional evaluation, hemorrhage volume and brain structure displacement). *P*-values < 0.05 were considered significant at a 95% confidence interval. Data were calculated using IBM SPSS 23 (Armonk, NY, USA), and the figures were obtained using GraphPad Prism 8 (San Diego, CA, USA).

### Ethics Statement

The experiments were conducted according to the Stroke Therapy Academic Industry Roundtable, RIGOR and HEADS recommendations (18, 29, 30) and the Animal Research: Reporting of *In vivo* Experiments guidelines (31) at our Neurological Sciences and Cerebrovascular Research Laboratory, La Paz University Hospital, Madrid, Spain. Animal care and experimental procedures were designed in accordance with our medical school's Ethical Committee for the Care and Use of Animals in Research (Ref. PROEX 296/16) according to the Spanish (RD 1201/2005 and RD53/2013) and European Union (EU) (86/609/CEE, 2003/65/CE, 2010/63/EU) rules.

## Results

### Clinical Status and Mortality

The functional status as well as the study variables in the experimental groups are shown in Table 1.

**Table 1:** Functional evaluation, ICH volume and CDR of the animals analyzed in the study.

		**Time**	**Healthy** **(***n*** = 10)**	**Sham** **(***n*** = 10)**	**ICH** **(***n*** = 11)**	***P***-**value**
Functional evaluation	Rogers [points (mean ± SD)]	Pre-ICH	0.00 ± 0.00	0.30 ± 0.95	0.00 ± 0.00	0.350
		48 h	0.60 ± 1.26	0.60 ± 1.26	3.54 ± 0.93	**0.001**
		4 d	0.30 ± 0.95	1.10 ± 1.45	3.64 ± 0.81	**0.001**
		1 mo	0.60 ± 1.26	0.80 ± 1.32	2.09 ± 1.81	0.065
	Beam walking [points (mean ± SD)]	Pre-ICH	0.00 ± 0.00	0.10 ± 0.32	0.18 ± 0.40	0.383
		48 h	0.00 ± 0.00	0.00 ± 0.00	4.64 ± 1.12	**0.001**
		4 d	0.20 ± 0.63	0.00 ± 0.00	3.45 ± 1.51	**0.001**
		1 mo	0.00 ± 0.00	0.00 ± 0.00	2.18 ± 1.83	**0.001**
	Tapered beam walking [% (mean ± SD)]	Pre-ICH	21.65 ± 6.68	20.16 ± 9.90	27.38 ± 13.88	0.376
		48 h	20.08 ± 6.91	23.70 ± 10.21	93.89 ± 15.57	**0.001**
		4 d	20.04 ± 9.42	16.01 ± 8.27	85.61 ± 21.70	**0.001**
		1 mo	16.60 ± 8.27	17.16 ± 7.31	68.53 ± 23.24	**0.001**
	Rotarod [s (mean ± SD)]	Pre-ICH	99.07 ± 33.86	97.73 ± 29.55	113.76 ± 9.79	0.613
		48 h	91.63 ± 46.73	107.00 ± 27.01	51.82 ± 27.79	**0.004**
		4 d	98.00 ± 40.74	107.57 ± 17.97	68.88 ± 31.37	**0.010**
		1 mo	91.47 ± 44.59	89.57 ± 36.93	69.82 ± 29.60	0.223
ICH volume	US (mm^3^ [mean ± SD])	5 h	–	–	58.92 ± 24.70	–
		48 h	–	–	66.22 ± 23.19	–
		4 d	–	–	65.27 ± 27.62	–
		1 mo	–	–	17.36 ± 9.97	–
	MRI [mm^3^ (mean ± SD)]	48 h	–	–	71.86 ± 24.75	–
		1 mo	–	–	21.49 ± 9.74	–
CDR	US (mean ± SD)	Pre-ICH	0.99 ± 0.02	1.01 ± 0.05	1.00 ± 0.00	0.587
		5 h	0.99 ± 0.02	1.01 ± 0.05	0.99 ± 0.04	0.401
		48 h	0.99 ± 0.02	0.99 ± 0.04	1.06 ± 0.04	**0.002**
		4 d	0.99 ± 0.02	1.00 ± 0.01	1.06 ± 0.04	**0.033**
		1 mo	0.99 ± 0.02	0.99 ± 0.03	1.06 ± 0.06	0.201
	MRI (mean ± SD)	48 h	0.98 ± 0.02	0.99 ± 0.03	1.06 ± 0.04	**0.001**
		1 mo	0.98 ± 0.01	1.00 ± 0.03	1.05 ± 0.07	0.096
Cisterns-dura mater distance	Contralateral side distance US [mm (mean ± SD)]	48 h	–	–	3.75 ± 0.30	–
		1 mo	–	–	3.44 ± 0.29	–
	Contralateral side distance MRI [mm (mean ± SD)]	48 h	–	–	3.84 ± 0.23	–
		1 mo	–	–	3.49 ± 0.26	–
	Ipsilateral side distance US [mm (mean ± SD)]	48 h	–	–	3.84 ± 0.41	–
		1 mo	–	–	3.64 ± 0.46	–
	Ipsilateral side distance MRI [mm (mean ± SD)]	48 h	–	–	4.09 ± 0.30	–
		1 mo	–	–	3.67 ± 0.45	–

*CDR, cistern displacement ratio; ICH, intracerebral hemorrhage; MRI, magnetic resonance imaging; US, B-mode ultrasound. Data were compared with Kruskal-Wallis test. Statistically significant values (p < 0.05) are in bold*.

A total of 2 rats died before the end of the study: 1 from the ICH group during the surgery and 1 from the healthy group during the MRI at 48 h.

### Intracerebral Hemorrhage Volume With B-Mode Ultrasound and Magnetic Resonance Imaging

**Figure 2:**
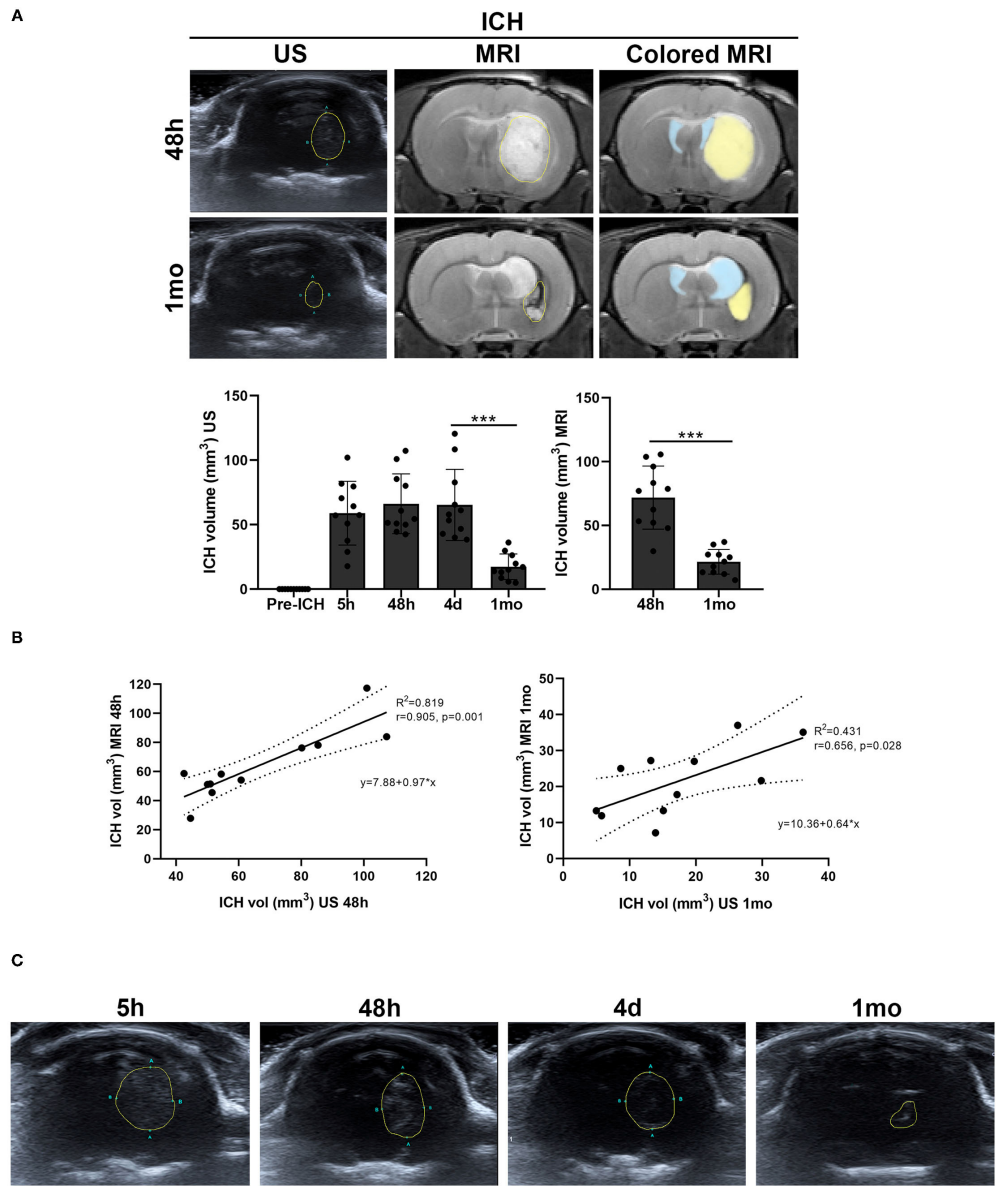
Hemorrhage volume of the ICH group measured by B-mode ultrasound and MRI (T2 images) **(A)**, and graphs of their correlation at 48 h and 1 mo **(B)**. Lateral ventricles are colored in light blue and ICH in light yellow in the MRI sections. **(C)** Representative images of the echogenicity of the cerebral hemorrhage over time. The hyperechogenicity of the hemorrhage was decreasing from 5 h after ICH induction until 1 mo. Data are shown as mean ± SD. ***p < 0.001. ICH, intracerebral hemorrhage; MRI, magnetic resonance imaging; US, B-mode ultrasound.

A decrease in the size of the ICH was observed throughout the study. By B-mode ultrasound, the ICH volume showed a significant decrease between 4 d and 1 mo (*p* = 0.001). Also by MRI, a significant decrease in ICH volume was observed between 48 h and 1 mo (*p* = 0.001) (Table 1, Figure 2A).

The measures obtained showed an excellent correlation between both techniques at 48 h (Pearson's correlation coefficient (*r*) 0.905, *p* = 0.001). At 1 mo after ICH, there was also a good correlation (*r* = 0.656, *p* = 0.028) (Table 1, Figures 2A,B).

In addition, it has been observed that the hyperechogenicity of the ICH at 5 h decreased over time until hypoechogenic signals were observed at 1 mo (Figure 2C).

### Brain Structure Displacement by Intracerebral Hemorrhage as Shown by B-Mode Ultrasound and Magnetic Resonance Imaging

**Figure 3:**
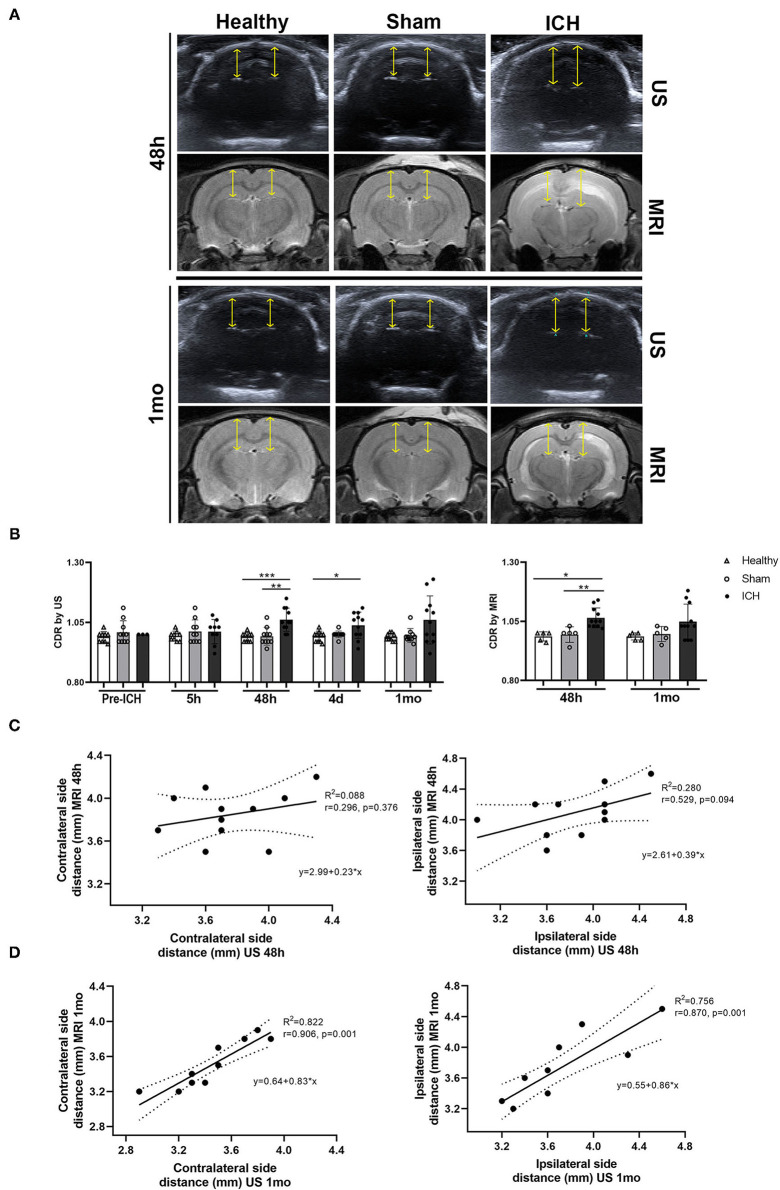
**(A)** Representative images of the subarachnoid cisterns identified by B-mode ultrasound and MRI (T2) in the different experimental groups at 48 h and 1 mo after ICH induction. Yellow arrows indicate the measured distance. **(B)** Quantification of the subarachnoid cisterns to dura mater distance ratio (CDR) between the different experimental groups by B-mode ultrasound and MRI. **(C,D)** Representative graphs of the correlation between B-mode ultrasound and MRI in the subarachnoid cisterns to dura mater distance on both the contralateral (left) and ipsilateral (right) sides at 48 h and 1 mo after ICH induction. Data are shown as mean ± SD. *p < 0.05; **p < 0.01; ***p < 0.001. ICH, intracerebral hemorrhage; MRI, magnetic resonance imaging; US, B-mode ultrasound.

By B-mode ultrasound, no differences were observed in the CDR between the healthy and sham groups pre-ICH (*p* = 0.422), at 5 h (*p* = 0.295), 48 h (*p* = 0.933), 4 d (*p* = 0.213) or 1 mo (*p* = 0.870) (Table 1, Figures 3A,B).

Pre-ICH, the CDR in the ICH group showed no significant differences compared with the healthy (*p* = 0.352) or sham groups (*p* = 0.788). Also, no differences were observed at 5 h between the ICH and the healthy (*p* = 0.192) and sham (*p* = 1.00) groups. However, there was an increase in the CDR in the ICH group at 48 h compared with the healthy (*p* = 0.001) and sham (*p* = 0.004) groups. At 4 d, an increase was found only between the ICH and healthy animals (*p* = 0.026); only a trend was found with the sham animals (*p* = 0.062). These significant differences were no longer present at 1 mo between the ICH group and the healthy (*p* = 0.118) and sham (*p* = 0.139) groups (Table 1, Figures 3A,B).

By MRI, the healthy and sham groups also showed no differences in the CDR at 48 h (*p* = 0.480) and 1 mo (*p* = 0.316). However, an increase in this CDR was found at 48 h in the ICH group compared with the healthy (*p* = 0.002) and sham (*p* = 0.008) groups. However, no differences were found between ICH and the healthy (*p* = 0.078) and sham (*p* = 0.111) groups at 1 mo (Table 1, Figures 3A,B).

After analyzing brain structure displacement, the correlation of this variable between the 2 imaging techniques was studied. At 48 h, a correlation of *r* = 0.296 (*p* = 0.376) was observed between B-mode ultrasound and MRI in the distance between the cisterns and dura mater on the side contralateral to the hemorrhage. The correlation that was found in the distance between B-mode ultrasound and MRI on the ipsilateral side was *r* = 0.529 (*p* = 0.094). Conversely, at 1 mo, the distance measured by both imaging techniques showed a correlation of *r* = 0.906 (*p* = 0.001) on the contralateral side and of *r* = 0.870 (*p* = 0.001) on the ipsilateral side (Table 1, Figure 3C).

## Discussion

In this study, we demonstrated the feasibility and accuracy of B-mode ultrasound imaging in the assessment of ICH in an experimental animal rat model, showing results that are equivalent to MRI in measuring ICH volume and secondary displacement of brain structures.

Although CT is the preferred tool for ICH diagnosis in patients with acute stroke (3, 32–34), MRI is typically used to monitor ICH in animal models (8). The ICH volume measured by the ABC/2 formula in T2^*^ MRI scans is closely associated with that measured in cryosections of mice (35). A good correlation between MRI and histopathological studies has also been demonstrated in an ICH model in rats (36). Concretely in rats, the signal of the hemorrhage by MRI (in T2) is hypointense, with isointense foci after a few hours (0–12 h) (36–39), corresponding to edematous areas (38). This hypointensity switches to hyperintensity after 24–72 h due to erythrocyte degeneration and cellular debris and is surrounded by a hypointense ring corresponding to neutrophils and macrophages (36–38). In agreement with these studies, we also observed the lesion as a hyperintense signal at 48 h, although the hypointense ring was not as clear in many of the images. This was probably due to the fact that the aggregation of neutrophils is maximal at 48 h; thereafter, the neutrophils disperse toward the center of the lesion to be replaced by macrophages, resulting in a less defined ring (38). The lesion remains hyper/isointense due to cellular debris and fluid-filled spaces with a hypointense ring of macrophages at 7 d (36–38). At 2–3 weeks and up to 28 d, the hematoma site consists of a fluid-filled cavity with an iso/hyperintense signal surrounded by a hypointense ring (macrophages) with dark areas corresponding to areas of necrosis and cavitations (36, 38, 40) equivalent to our images at 1 mo.

To our knowledge, the 1981 study performed by Enzmann DR et al. was the first study evaluating ICH with ultrasound in an animal model (41). By introducing a hematoma into the parietal lobe by craniotomy in dogs, they were able to identify a highly echogenic hemorrhagic lesion in acute ICH. This signal became hypoechoic with echogenic borders at 3–4 d due to the loss of erythrocyte integrity (41). Between days 9 and 13, the formation of a network of collagen and macrophages has been described in the animal model, which would give rise to a hypodense ring around the hematoma that later narrows due to the increase in the connective tissue capsule (42). This data highlights the lack of recent preclinical scientific articles on ultrasound in ICH. Previously, it had been shown that ultrasound does not induce additional damage when applied to the ICH. In particular, Stroick et al. had demonstrated that the effects of diagnostic ultrasound with microbubbles did not cause more brain damage in ICH rats (43). Also, Ke et al. used high-frequency transcranial Doppler ultrasound to study blood flow velocity after ICH in rats (17). Apart from these studies, to our knowledge, there are few studies that have examined ICH using ultrasound. Therefore, the present study would be one of the first to study ICH and monitor it by means of ultrasound in an animal model. Here, we were able to identify a hyperechogenic mass in the brain parenchyma of animals at 48 h that was less defined at 1 mo due to its transformation into a more hypoechoic signal, correlating with images obtained in patients (12, 42).

We have also demonstrated that B-mode ultrasound and MRI detect a very similar ICH volume in early states, with a significant correlation between both techniques. At late stages, we continued to observe a significant correlation in the measurement of ICH volume by both techniques but lower than that obtained at 48 h after ICH. The decrease over time of ICH volume (44, 45), attenuation and resolution of the hematoma as a fluid-filled or slit-like cavity (45, 46), the narrowing of the echo-dense seam shown at the last stage on ultrasound (42) and the heterogeneity in the MRI signal could hinder visualization and measurement, thus explaining this lower correlation at the late stage. The clinical application of ultrasound had already been demonstrated for the diagnosis of ICH in 1 or a maximum of 35 patients (42, 47–50). However, further studies are needed to establish the diagnostic value of ultrasound and its accuracy to guide therapeutic decisions in ICH (12). The small number of participants, short study times and the lack of preclinical experimentation highlight the need to continue studying this technique to be able to implement it with MRI or CT assisting in the monitoring of ICH.

In parallel with the study of ICH volume, we also analyzed the possible displacement of brain structures as a consequence of ICH. ICH causes displacement of adjacent structures due to mass effect in the acute phase (51). In later phases, brain atrophy occurs with atrophy of the caudate and enlargement of the ipsilateral ventricle (52, 53). In a previous study, we had demonstrated the ability of B-mode ultrasound imaging to identify fluid-filled cavities and to differentiate them from brain tissue in rats (26). We have also identified the subarachnoid cisterns as the main structures that can be observed. These cisterns are filled with cerebrospinal fluid and connect the ventricular system and the subarachnoid space (26, 54). In this study, we observed a displacement of these cisterns, specifically on the ipsilateral side, with relation to the dura mater that could be explained as a late consequence of mass effect. In addition, we observed a high correlation in the measurement of the subarachnoid cisterns distance between B-mode ultrasound and MRI, emphasizing again the similarity in measurements between the 2 techniques.

Although ultrasound allows us to observe the mass effect derived from ICH, it does not identify important components of the lesion, such as edema (18); it also has the disadvantage of being operator dependent and has limitations in image acquisition due to bone or gas-filled structures (14). However, its advantages and the results presented in this study demonstrate its ability to be successfully employed in rats.

In conclusion, we have shown an excellent correlation between the 2 imaging techniques that shows B-mode ultrasound to be a tool as accurate as MRI in the assessment of ICH lesion volume and displacement of brain structures. B-mode ultrasound's ability to measure the volume of hemorrhage and the displacement of brain structures provides a novel approach for the monitoring of ICH in animal models. Despite the need for further studies, these findings support the use of B-mode ultrasound for monitoring ICH in future preclinical and clinical studies.

## Data Availability Statement

The raw data supporting the conclusions of this article will be made available by the authors, without undue reservation.

## Ethics Statement

The animal study was reviewed and approved by Neurological Sciences and Cerebrovascular Research Laboratory, La Paz University Hospital, Madrid, Spain. Animal care and experimental procedures were designed in accordance with our medical school's Ethical Committee for the Care and Use of Animals in Research (Ref. PROEX 296/16) according to the Spanish (RD 1201/2005 and RD53/2013) and European Union (EU) (86/609/CEE, 2003/65/CE, 2010/63/EU) rules.

## Author Contributions

MG-dF, GR-A, and MG-F: manuscript writing. MG-dF, IG-S, and FL-G: methodology and investigation. MG-dF, IG-S, FL-G, LD, LO-O, MAdL, BF, MG-F, ED-T, and GR-A: manuscript revision, reading, and approval of the final manuscript. ED-T, GR-A, and MG-F: funding acquisition, supervision, and project administration. All authors contributed to the article and approved the submitted version.

## Funding

This work had grant support from the Spanish Ministry of Health—Carlos III Health Institute (ISCIII) and the European Regional Development Fund (FEDER Funding) with PI16/01052 project, the INVICTUS PLUS network grant (RD16/0019/0005), Miguel Servet (CP15/00069, CPII20/00002 to MG-F; CP20/00024 to LO-O), a predoctoral fellowship (FI17/00188 to MG-dF; FI18/00026 to FL-G).

## Conflict of Interest

The authors declare that the research was conducted in the absence of any commercial or financial relationships that could be construed as a potential conflict of interest.

## Publisher's Note

All claims expressed in this article are solely those of the authors and do not necessarily represent those of their affiliated organizations, or those of the publisher, the editors and the reviewers. Any product that may be evaluated in this article, or claim that may be made by its manufacturer, is not guaranteed or endorsed by the publisher.
